# From scarcity to ultra-processed foods: a conceptual analysis of how their properties interact with characteristics of food intake regulation mechanism

**DOI:** 10.3389/fnut.2026.1803885

**Published:** 2026-04-10

**Authors:** Filip Oosterlinck, Yves Segers

**Affiliations:** Interfaculty Centre for Agrarian History, KU Leuven, Leuven, Belgium

**Keywords:** energy density, engineered sensory reward, food intake regulation mechanism, hyperpalatability, ultra-processed foods, UPF product attributes

## Abstract

Since the mid-twentieth century, there has been a rapid rise in the production and consumption of ultra-processed foods (UPFs) so that they now contribute up to 60% of dietary energy intake in several Western countries. Existing accounts emphasize economic, political, food-system, and socio-cultural drivers of this shift. In this conceptual article, we discuss whether from a historical perspective, UPFs are a unique category of foods or rather an intensified extension of a long-standing trajectory of humans using various methods, such as mechanical processing, fermentation and cooking, to improve the digestibility, energy availability and palatability of their foods. Hereto we identify the typical properties used to explain the appeal of UPFs, distinguishing (i) direct intake drivers: engineered palatability, food matrices that enable high eating rates, high energy density and high sensory variety and (ii) market-mediated product attributes: convenient and portable formats, low effective cost, branded and marketed, ubiquitous availability, and long shelf life. We show that for none of these, UPFs form a clearly distinct grouping, but that what sets them apart is that on average they score higher on these properties, and they combine more of them at the same time. We then propose a conceptual framework to assess whether UPFs are unique in the way that they appeal to our evolved food intake regulation system. Hereto we map the properties onto (quantifiable) characteristics of the food intake regulation system in a testable framework. We conclude that a property-based assessment of UPFs suggests them to be an extension, rather than a categorical break from historical traditions, and that understanding the contemporary prevalence of UPFs requires explicit consideration of their interaction with evolved food intake regulation mechanisms, alongside established structural explanations, and we outline implications for future empirical tests and multi-level interventions.

## 1 Introduction

The potential links between the increasing consumption of ultra-processed foods (UPFs) and the rise of obesity and non-communicable diseases preoccupies academic and popular nutrition and medical sciences (1–4). Reflecting this concern, the most recent edition of the U. S. government’s nutrition guidelines (2025–2030) explicitly recommends limiting the intake of highly processed foods (5).

Several prior studies have tried to explain the growth of the consumption of UPFs. For example, Béné et al. (6) reviewed food system drivers albeit without a specific focus on UPFs. Wood et al. (7) employed system-thinkings approaches to map the global rise of ultra-processed foods in population diets and developed a causal loop diagram (CLD) of the global UPF system, while Slater et al. (8) used network analysis to examine the concentration of corporate power within the UPF sector. Godsey et al. (4) focused on behavioral drivers of consumption and Hall (9) discussed typical properties. Collectively, these analyses offer valuable insights into economic, political, and structural determinants of UPFs consumption.

What remains largely unanswered is the question whether, from a historical perspective, ultra-processed foods are a unique category of foods or rather represent an extension of a long-standing trajectory of humans using various methods, such as mechanical processing, fermentation and cooking, to improve digestibility, energy availability and palatability of their food: in other words do we need to see them as a categorical break with the past, or are they rather an intensified development of human efforts to select and prepare food. In this article we propose a conceptual framework to answer that question. Hereto we first identify a set of properties that are typically associated with UPFs and are argued to make them appealing to consumers, and we assess whether these properties are unique to UPFs. We then identify the main characteristics of our evolved food intake regulation systems and propose a testable framework to evaluate the interaction of the common properties of UPFs with these characteristics.

By detailing these interactions, we situate the modern rise of UPFs consumption in the context of our species’ evolutionary adaptations to for instance food scarcity and predation pressure. Humans have long modified foods through practices such as mechanical processing, fermentation and cooking, progressively increasing digestibility and energy density.

The article is structured as follows: First, we describe the concept of ultra-processed foods and identify both product-level characteristics and broader drivers of UPFs consumption. We then evaluate the existing experimental evidence on whether these properties drive food intake, and on whether they are unique to UPFs. Next, we present relevant human food intake regulation mechanisms and their evolutionary origins. Finally, we propose an integrative conceptual framework linking the properties of ultra-processed foods to the characteristics of food intake regulation mechanisms. The aim is not to offer an exhaustive treatment of either UPFs consumption drivers or evolutionary eating regulation, but to articulate and present a conceptual framework that can guide future empirical and theoretical work. We do not review or discuss the myriad publications that address the (potential) health effects of ultra-processed foods but refer to some recent reviews (10–13).

## 2 Ultra-processed foods and the main drivers for their consumption

### 2.1 Ultra-processed foods

The concept of ultra-processed foods UPFs was originally developed by Monteiro and co-workers (14, 15). Later rebranded as NOVA, it is now composed of 4 categories, with the first group being minimally or unprocessed foods and the fourth NOVA group being that of ultra-processed food and drink products (16): “These are industrial formulations typically with five or more and usually many ingredients.” Or in other words, foods that are extensively processed and contain numerous additives, preservatives, and manufactured ingredients. The main categories include confectionery, soft drinks, breakfast cereals, salty snacks (such as potato chips), industrial prepared pizza, French fries, cakes and cookies, and reconstituted meat (17, 18).

Currently in the U. S. and U. K., UPFs consumption is estimated to be around 55–65% of dietary energy and this has stayed fairly constant since the turn of the century (19, 20).^1^ For instance, based on an analysis of the 2017–2018 National Health and Nutrition Examination Survey (NHANES) data Steele, O’Connor (17) concluded that the energy contribution of UPFs was 58.2% ± 0.9% of the total energy. U. K. and U. S. UPFs consumption is among the highest levels in the world, but the rest of the world is catching up (21).

The NOVA classification is debated: healthy items can be UPFs and vice versa (22–24), there is an overlap with existing nutrient indices (25), and some argue it is the ultra “formulation” rather than the processing that is the main problem (23). More broadly, one could argue that all foods are to some extent processed, since processing is needed to ensure digestibility, shelf life, safety, palatability, and appeal (24). Hence, in this article we evaluate whether NOVA ultra-processed foods are a unique food category by considering their common properties (see next section), and their interaction with our evolved food intake regulation mechanisms (see Sections 3 and 4).

### 2.2 Main factors driving the consumption of ultra-processed foods

**Figure 1:**
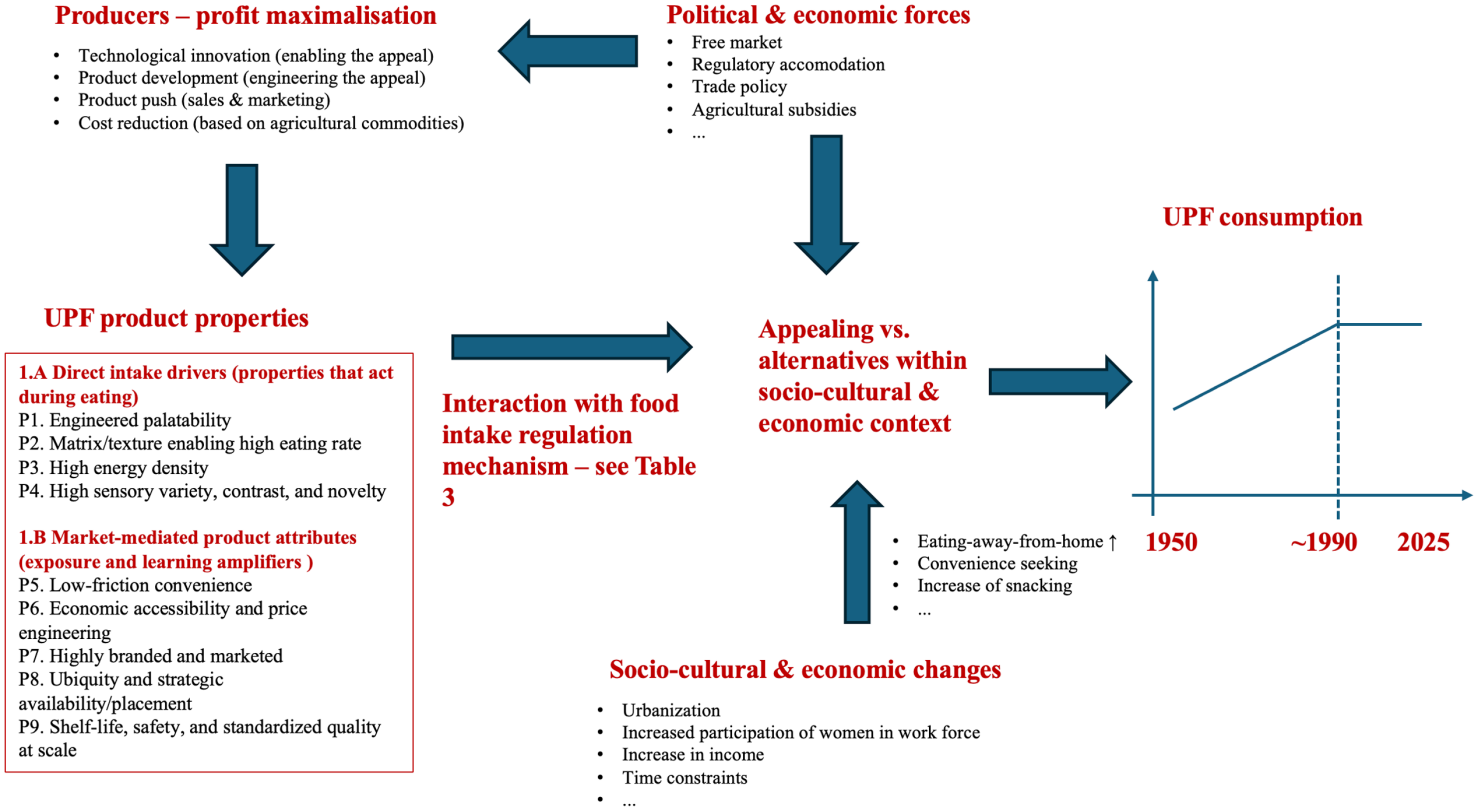
Potential drivers that explain the increasing consumption of ultra-processed foods UPFs. Synthesized from extant literature: for the overall scheme work by Béné et al. (6) on food systems drivers, Wood et al. (7) on causal loop diagram (CLD) of the global UPF system, Slater et al. (8) on a network analysis of UPFs, National Food Strategy (108) for the junk food cycle. For the specific characteristics of UPFs explaining their appeal, work by O’Connor et al. (26) on potential pathways that properties of UPFs promote excess energy intake as well as by Hall (9) who gives a list of UPFs characteristics and Hall et al. (33) and some other sources (4, 9, 24).

Several recent reviews have discussed the main characteristics that define the appeal of UPFs, and the main drivers underlying the growth (4, 7, 8, 24). Figure 1 gives a conceptual overview of these different drivers which are active at different levels.

Starting at the lowest level, ultimately it comes down to the fact that individual consumers buy ultra-processed foods (versus minimally or non-processed foods). Hence, the properties of UPFs must be appealing to consumers and that is the focus of this section. Before discussing these properties, it is important to note that at higher levels, there are manifold structural drivers underlying the increasing UPFs consumption such as economic ones (e.g., corporate power and amount of marketing), socio-cultural ones (increasing time scarcity, snacking, …) and political levels ones (e.g., agricultural subsidies, etc.), but these are out of scope of the current article and the reader is referred to the literature (4, 7, 8, 24).

Based on the existing literature we identified a set of typical UPFs properties: see Figure 1 for an overview and the connection to the wider set of drivers, and Table 1 for the available experimental evidence substantiating their effect, a proposed measurable proxy, and an indication for how unique the individual properties are to the UPFs category. Before discussing each of them in detail, it can be observed in general that for none of the properties typically associated with UPFs, there is a sharply defined boundary/threshold above which foods are part of the ultra-processed category and below which foods are part of the non or minimally processed foods category. Rather, on average the UPFs category scores higher on each of the individual subsets, and foods from the UPFs category combine several of these properties at once. We will come back to this point at the end of the section.

**Table 1:** Overview of most common UPFs product features, a proxy for their quantification, experimental evidence that they increase Food Intake.

Common UPFs product feature	Proposed proxy for quantification	Current experimental evidence that feature increases Food Intake	Unique to UPFs?	Open research questions
P1. Engineered palatability	Palatability score based on nutrition content (31) for overall scoring and one based on hedonic perception for deep-diving	Food intake increases with higher palatability in individual test meal based studies (27), a food dairy based study (28) and two inpatient feeding studies lasting 28 continuous days (38)	No, but on average UPFs seem to be higher in palatability at least based on nutrient content (19)	- Evaluate whether nutrient content scores need to include flavorings and other engineered ingredients
P2. High eating rate/soft texture	Eating rate (kcal/min)	Higher eating rate has been shown to lead to higher food intake in an RCT (33), and two inpatient feeding studies (33, 38)	No, but on average UPFs have a significantly higher eating rate (32)	- Clarify underlying reason for higher eating rate (soft texture, …)
P3. High energy density	Energy density (kcal/100 g) (19)	Energy density has been shown to lead to higher food consumption in short term laboratory studies (38, 39, 109)	No, but on average UPFs have a significantly higher energy density (37)	- Confirm effect of ED with long term naturalistic studies
P4. High sensory variety, contrast and novelty	# of distinct product lines and SKU’s	Increased variety has been shown to increase food intake in both animal and human short term laboratory studies (42)	No, but given the amount of variety in common UPFs (45) they on average show higher variety	- Confirm effect of variety with long term naturalistic studies
P5. Convenience / low friction	Convenience scale proposed by Chandon and Wansink (110)	Stockpiled high convenience products are consumed faster in study using household scanner data and in a field study with 60 households (110)	No real data available, but there are convenient unprocessed foods	- Quantify convenience of UPF vs. non UPFs - Confirm effect with long term naturalistic studies
P6. Economic accessibility and price engineering	$/100 kcal [see, e.g., (37, 111)]	Two systematic reviews concluded that price increases/decreases increase/decrease food consumption of individual categories (112, 113) Higher food insecurity leading to higher UPFs consumption (50) and lower diet cost for higher UPFs consumption (37, 49)	No, but on average UPFs have a significantly lower per calorie cost (37)	- Confirm effect with long term naturalistic studies
P7. Branded, marketed,	Advertising dollar expenditure	Evidence that banning fast food advertising reduces consumption in study using Quebec’s fast food ban (62) and systematic review confirming effect of marketing on consumption (61)	No, but marketing spend on UPFs is significantly higher (55, 61)	- Confirm effect with long term naturalistic studies
P8. Ubiquity and strategic availability/placement	Availability density	No hard data found that shows increasing availability drives demand in absolute sense. Quite some evidence that UPFs are readily available but no hard evidence mechanistically linking the ubiquitous presence and consumption	No, but data suggests that (UPFs) snack foods are more available in non- food stores (64)	- Quantify availability of UPFs vs. non UPFs - Confirm effect with long term naturalistic studies
P9. Shelf-life/safety/standardization	Ambient shelf life	Stockpiling, enabled by long shelf life, has been shown to increase food consumption in study using household scanner data and in a field study with 60 households (110)	No, but ambient shelf stability is easier to achieve with UPFs (24)	- Quantify shelf life of UPF vs. non UPF - Confirm effect with long term naturalistic studies

The properties presented in Table 1 and discussed below differ in when and how they exert their effects. Some features act primarily during consumption (e.g., engineered palatability, high eating rate, energy density and variety), potentially shaping reward intensity and the timing/strength of satiation and satiety feedback. Other features act primarily before and between eating episodes (e.g., convenience, low cost, branding and marketing, availability/placement, and shelf life), potentially increasing exposure to cues, lowering acquisition and consumption effort, and strengthening reinforcement learning and habit formation.

For analytical clarity, we therefore group UPF product features into (i) direct intake drivers (1A in Figure 1) and (ii) market-mediated product attributes (1B in Figure 1). Among direct intake drivers, UPFs frequently combine (P1) engineered palatability (e.g., fat–sugar–salt combinations and engineered flavor/texture systems), (P2) food matrices that reduce oral processing demands and enable high eating rates (“fast calories”), (P3) relatively high energy density, and (P4) high sensory variety and contrast that facilitates switching and reduces sensory-specific satiety.

Discussing each of these in turn: According to O’Connor et al. (26) palatability (P1) is the positive hedonic evaluation of a food’s sensory characteristics (i.e., taste, smell, texture, temperature, visual appearance, sound and trigeminal input). Palatability is not a static feature of a food/beverage; it changes in response to sensory monotony and metabolic need state. Food intake has been shown to increase as palatability goes up (27, 28). UPFs frequently contain high amounts of salt, sugar and fat so that in general UPFs elicit higher approach motivation than unprocessed and minimally processed foods (29). Two main approaches have been followed in literature to quantify palatability: One uses human’s hedonic perception and the other is based on ingredient composition. The former measures of palatability include the perceived pleasantness of a given food, intent to eat, and the amount of food consumed (27, 30). Even though this is linked to the actual perception of humans, this way of quantification is limited by the fact that only a limited set of products can be evaluated at the same time, and it is thus difficult to make an overall judgment whether UPFs are more palatable or not. The second quantification method is based on the relative amounts of certain ingredients such as fat or sugars. This makes an overall comparison more straightforward. E.g. Fazzino et al. (31) developed a quantitative definition for hyper-palatability HPFs by extracting common HPFs descriptive definitions from the literature and using nutrition software to quantify ingredients of fat, simple sugars, carbohydrates, and sodium. HPFs from the literature aligned with three clusters: (1) fat and sodium (>25% kcal from fat, ≥0.30% sodium by weight), (2) fat and simple sugars (>20% kcal from fat, >20% kcal from sugar), and (3) carbohydrates and sodium (>40% kcal from carbohydrates, ≥0.20% sodium by weight). In a subsequent article Fazzino and coworkers (19) then evaluated the prevalence of ultra-processed, hyper-palatable, and high energy density foods, observing that in the U. S. between 1988 and 2018 UPFs and HPFs constituted about 60% of foods consumed, with about 68–78% of UPFs also being HPFs, and HPFs being about 70% UPFs. So even though hyper-palatability is not exclusively limited to UPFs, in general the latter score relatively high.

Many UPFs have a softer texture that enables a higher eating rate (P2) and in general UPFs have a higher energy density (P3) than non - or minimally processed foods (32). In a recent random controlled trial by Hall et al. (33) the energy intake rate in the group eating UPFs (48 kcal/min) was >50% higher than in the unprocessed group (31 kcal/min) and this indeed was correlated with a higher overall energy intake. Forde et al. (32) further confirmed this observation that across a wide sample of foods, those classified as UPFs had, on average, a faster energy intake rate (kcal/min) than unprocessed foods, although there was significant heterogeneity within each processing category. For the set of foods that they considered, the average energy intake rate for unprocessed foods was 35.5 kcal/min and that for UPFs was 69.4 kcal/min. For instance the rate for unprocessed foods ranged from 2 kcal/min (iceberg lettuce) to 230-240 kcal/min (fresh full-fat milk, orange juice), whereas that for UPFs ranged from 0 kcal/min (Cola Light) to 9 kcal/min (powdered vegetable soup) up to 249 kcal/min for chocolate semi-skimmed milk (32).

A potential explanation for this heterogeneity within categories, is the influence of texture with Teo et al. (34) showing that softer texture foods are eaten at higher eating rate for larger total consumption than hard textured foods, both for minimally and ultra-processed foods. Historically speaking, the comparatively reduced amount of fiber present in UPF dominated diets might be an important reason: the average fiber content of the late Paleolithic diet is estimated to be at ~ 45 g versus only about 20 g in the 1970’s U. S. diet (35).

With regards energy density (P3), the food only (excluding beverages) energy density of the Paleolithic diet is estimated to be in the order of 1.35 kcal per gram (35), whereas in the U. S. it is in the order of 1.85 kcal per gram (in 1995) (36), and for ultra-processed foods it is on average 2 kcal/g (37). Energy density has been shown to lead to higher food consumption in short term laboratory studies, but it still needs to established whether this is also the case in the long term natural situation (38–40).

Increased variety (P4) has been shown to increase food intake (27, 41), with variety acting within a meal, across meals, and over longer periods, i.e., dietary variety (41–43). The food industry has evolved foods with elaborate adaptations such as tofu turkeys, sugar-free products that taste sweet or fat-free foods with fatty mouthfeel (44). And this has led to increasing variety of ultra-processed foods: for instance between 2002 and 2009, ~2000 new SKU’s and ~254 new product lines of frozen pizza were introduced in the U. S., 3,115 new SKUs and ~612 new product lines of cold cereal, ~12,878 new SKUs and ~1,626 new product lines of salty snacks (45).

In addition to these direct drivers, many UPFs also have market-mediated properties that enhance their appeal, including (P5) low-friction convenience and portability, (P6) economic accessibility and price engineering (often reinforced by promotions and portion-sizing), (P7) branding and marketing, (P8) ubiquitous availability, and long shelf life (P9) and standardized quality that enable constant availability and consistent sensory reward. We will now discuss these in more detail.

Many ultra-processed foods are manufactured to be ready-to-eat (RTE), requiring no preparation before quick, easy consumption (46, 47) providing low-friction convenience (P5). Ultra-processed are generally also relatively low-cost (P6), especially per unit of energy provided: cost level of UPFs is in the order of 0.4–0.6 $/100 kcal in the period of 2004–2016, whereas unprocessed foods were at 1–1.4 $/100 kcal (37). UPFs make use of cheap, standardized commodities, such as refined cereal flours, sugar, fats and oils (23). This relative cost advantage also applies to individual categories: for instance canned vegetables largely have lower costs per edible cup compared with frozen and fresh (48). Work by Gupta, Rose (49) shows that the percentage energy from UPFs was inversely linked to lower food expenditures and diet cost, confirming the influence of the relative economic affordability (P6) of many UPF foods. Moreover, Leung et al. (50) show that higher food insecurity leads to higher UPF consumption, and reason that low-income adults may selectively seek out ultra-processed foods to ensure sufficient food for their household during food-related hardship.

The role of marketing and branding (P7) seems a key element in the appeal of many UPFs (51–53). In the U. S. the advertising spend was ~2% of the food marketing bill in 1960, rising to about 4% in 1990 (54). Individual companies spend huge sums on promoting their wares. For instance, advertising spend of U. S. food and beverage companies was ~7 billion USD in 1997, of which breakfast cereal manufacturers spend around 792 million USD, soft drink manufacturers 549 million dollars whereas fruit and vegetables producers no more than 105 million dollars, and the USDA spend just 330 million dollars on nutrition education (55). The outcome is that children are inundated with advertisements on television (56–60). The most common categories of food products promoted to children are pre-sugared breakfast cereals, soft drinks, savory snacks, confectionary and fast foods, with estimates for the proportion of these products varying from 60 to 90% (61). Dhar and Baylis (62) used Quebec’s ban on fast-food advertising as a natural experiment and observed a 13% lower purchase propensity for those affected by the ban. Veerman et al. (63) considered the effect of limiting TV food advertising on obesity and concluded it could significantly lower obesity levels by reducing consumption of energy-dense foods.

Another market-mediated product attribute is the ubiquity of ultra-processed foods (P8), which are widely available (9), even in non-food retail stores: Farley et al. (64) observed that in a survey of U. S. stores, snack food was available in 41% of the stores; the most common forms were candy (33%), sweetened beverages (20%), and salty snacks (17%). In some areas, so-called food deserts, highly processed foods are sometimes the only foods available (65). However, there is not a lot of hard evidence confirming that presence of UPFs drives consumption. In their 2012 review on the local food environment and diet, Caspi et al. (66) found moderate evidence in support of the causal hypothesis that neighborhood food environments influence dietary health, but they also pointed out reproducibility and more in general methodological issues. Specifically, the evidence for fast food outlets and fast-food consumption was the weakest, perhaps due to a relative ubiquity of fast-food outlets compared to other food sources. In a 2016 study, Thornton et al. (67) performed what they called a natural experiment and found no evidence that the overall consumption of McDonald’s products increased among local residents in the 12-month period after the opening of a new McDonald’s restaurant. So, there is quite some evidence showing that UPFs are omni-present but no real hard evidence showing a mechanistic link between the presence and the consumption, presumably because this is experimentally quite hard to show.

Ambient shelf stability (P9) is easier to achieve with high-energy, low-moisture foods with high carbohydrate and fat content (24), so many UPFs have a long shelf life.

Based on this analysis and Table 1, one can conclude that for the properties that are commonly associated with ultra-processed foods and that are used to explain their appeal, in fact UPFs do not form a distinct category separated by a hard threshold level. Rather, in general, UPFs rate relatively higher on average for these properties, significantly for most of them. This is in line with the view that ultra-processed foods are an extension of the long-standing trajectory of humans processing their food. Arguably, what sets UPFs apart is that they combine most (if not all) of the properties listed in Table 1 at the same time, whereas minimally and non-processed foods might rate high in one or two of them but will not combine them all simultaneously (supporting evidence for this point is provide by Supplementary Table S1).

To further shed light on this, it is instructive to evaluate the interaction of the listed properties with our innate food intake regulation mechanisms: this could clarify whether the fact that UPFs combine many appealing properties simultaneously is the key driver for their appeal or whether there is still a material difference in their interaction with our eating control systems. Hereto we will now build a framework to map the properties of Table 1 to our innate food intake control system. We will first identify some key (quantifiable) characteristics of the food intake regulation mechanisms in the next section. Then we will propose a hypothetical mapping of the listed properties unto those characteristics, allowing further work to reveal whether UPFs indeed materially differ in their interaction with our innate food intake control system biasing relative intake toward higher UPF consumption.

## 3 Evolved food intake regulation mechanisms

As explained in the previous section, we now shift our focus inward to identify (quantifiable) characteristics of our innate food intake regulation systems, so that we can subsequently evaluate their interaction with the properties commonly associated with UPFs defined in Table 1. Several recent reviews have dealt with our eating control mechanisms and the underlying evolutionary mechanisms, focusing either on the overall physiological control of eating (68), the influence of the foraging vs. predator avoidance trade-off (including insights from the field of behavioral ecology) (69), the importance of the gut-brain communication (70), …. Not everything is fully understood and given the complexity the literature is still somewhat scattered. Since it is problematic to directly link the previously defined properties to the actual neurological, physiological… regulatory systems, we rather propose to identify the key characteristics to evaluate the influence of the UPFs properties. Based on the available literature we have identified a set of 7 characteristics of human’s food intake regulation system (M1-M7 see Table 2) that we think are most relevant to how the common properties of UPFs can bias intake.

**Table 2:** Overview of the identified food intake control system characteristics and proposed proxies for quantification.

Food intake regulation system characteristic	Description	Proposed proxy for quantification
M1. Drive for efficient foraging	Bias toward higher net energy gain per unit time/effort, including preferences for low-acquisition-cost foods and reduced oral processing costs (chewing, swallowing, digestion)	Nothing readily available in literature. Proposed non validated: Energy gain per cost, with cost composite value of monetary, time and digestion cost
M2. Preference for salt, sweet…	Approach biases for cues historically associated with carbohydrate (sweet), protein (umami), sodium (salt appetite), and fat-associated cues, alongside avoidance and disgust responses to likely toxins/pathogens (e.g., bitter/putrid cues)	Nothing readily available in literature. Proposed non validated: nutrient score composed of fat, sodium and sugar content
M3. Hedonic influence	Hedonic valuation (liking), incentive motivation (wanting), cue-triggered desire, and habit formation shaped by repeated pairing of cues/contexts with reliable post-ingestive reward	*Should minus want* score as devised by Milkman et al. (86)
M4. Time-lag of satiation/satiety	Within-meal and post-ingestive stop-signals (gastric, hormonal, neural) operate with time lags; rapid calorie delivery can outpace these brakes, especially under distraction	Postprandial feeling of fullness and hunger in function of time per 100 g ingested [building on Gibbons et al. (90)]
M5. Preference for dietary flexibility	A tendency to diversify intake (reducing deficiency risk under uncertainty) and to show declining pleasantness for repeated sensory exposure within a meal, promoting switching and higher total intake when variety is available	Nothing readily available in literature. Proposed non validated: perceived variety (by human panel)
M6. Asymmetric energy regulation	Stronger defense against energy deficit than against surplus, creating a bias toward storing energy during periods of abundance and making chronic small surpluses easier to sustain	Nothing readily available in literature. Proposed non validated: energy density
M7. Social modulation and social learning	Social facilitation of eating and sensitivity to norms/prestige cues that shape what is perceived as desirable, appropriate, and rewarding to consume	Nothing readily available in literature. Proposed non validated: perceived societal acceptance score

These seven characteristics (M1–M7) will form the lens through which we will examine the influence of UPFs properties in Section 4.1, where we will proceed to map the specific features of UPFs onto these characteristics in an integrative framework. In the following, we discuss them each in more detail.

Ninety-eight percent of hominid existence has been shaped by hunting and foraging with selection for cognitive and behavioral repertories, nutritional requirements and physiological patterns adapted to harsh environments with fluctuations in food availability, food shortages and periodic high energy expenditures, potentially toxic foodstuffs and mostly (at least at first) under the continuous threat of predation (44, 71). Humans are clear evolutionary outliers for the amount of time spend feeding (excluding nonfeeding components, such as searching for food), i.e., spending an order of magnitude less time than predicted by phylogeny and body mass (72). There are a few fundamental differences that separate the human masticatory apparatus and digestive tract from that of other related primates: a diminution of the human mouth, jaw muscles, jaw, incisors, molars, stomach, colon and an enlargement of the small intestine relative to the overall size of the gut (73, 74). The reduction in masticatory apparatus and colon size relative to the other great apes, means humans have a greatly reduced capacity to consume and digest high quantities of fibrous or difficult-to-digest foods. Digestive rate, along with differences in the gut proportions for humans relative to other great apes (high-volume small intestine and low-volume colon) clearly indicate that the human dietary strategy shifted at some point in our evolutionary history away from processing copious amounts of low-quality, nonfermentable fibrous plant foods, and instead focused on higher quality, easily chewable, digestible, and easily fermented foods (73, 74).

The evolutionary origins of these “easier-to-digest” foods are still debated, but are likely produced by a combination of food processing (slicing, pounding, cutting, peeling, scraping, skinning, grinding and sun drying, … (73, 75–77)), cooking (74, 78), increased meat consumption (79) especially of high-fat meat and marrow (74), shift in plant diet, use of fermentation (80)… Food processing can be seen as a technological way of externalizing part of the digestive process (81) and this likely co-evolved with our taste and gustative evolution.

These all tend to lower the (relative) masticatory and digestive effort, significantly augment nutritional accessibility at all stages of digestion (enhancing the absorptive capacity of the small intestine as well as increase the metabolic power of the gut microbiota) (73), and increase the palatability of raw food (82). Thus, a first important characteristic is that humans optimize foraging efficiency by decreasing the cost of food gathering and digestion (M1) by the various means just discussed. A logical quantification proxy here could be the total cost per ingested kcal.

The human gustatory system functions as a critical nutritional gatekeeper (M2), evolved to solve the omnivore’s dilemma of identifying safe energy while avoiding environmental toxins (83). Taste is an especially important sense for omnivorous species given that the potential range of foods, their variation in nutrient content, and the hazards of accidental toxin ingestion increase with the variety and complexity of the feeding strategy. Humans have a preference (M2) for sweet, sour, salty, fatty, umami, and starchy foods (83). A potential quantification proxy could be a nutrient score based on fat, sugar and salt content.

Eating when energy reserves are depleted and abstaining from eating when replete serves a ‘homeostatic’ model for the regulation of energy balance. Non homeostatic `hedonic’ eating (M3) refers to the involvement of cognitive, reward, and emotional factors, and it is not regulated or compensated by some form of metabolic feedback (84). In the fasted state both the homeostatic and hedonic system tend to promote food intake in agreement with both energy and reward needs, whereas post-prandially, consumption of food in the absence of hunger might continue because of the reward system (85). With regards hedonic eating, food reward has classically been analyzed in terms of liking and wanting. These are represented in the brain in distinct but overlapping areas (85). Milkman et al. (86) defined a *should minus want* score capturing that people frequently *hedonically* want foods, linking them to immediate short-term benefits, while consumers know they are not part of the *should* foods which would suit people’s longer term interests better. And this seems an appropriate quantification proxy.

Metabolic or homeostatic eating is regulated by a complex combination of systems which take a certain time (M4) and can thus potentially be overridden by a too fast ingestion of calories. Satiation is the short-term inhibition of within-meal food intake that develops during a meal from the cumulative effects of inhibitory signals generated by the ingestion of food items (I can no longer eat anything), and satiety is the inhibition of food intake during the inter-meal period, whereby sensory and cognitive processes interact with post ingestive and post absorptive peripheral and central mechanisms (68, 87). This metabolic eating regulation system shows biological degeneracy: i.e., structurally different elements eliciting the same response, and it has been argued that this it is an inevitable product of natural selection (88), and also helps in biological robustness, that is, maintenance of function despite internal and external perturbation (89). In the case of cessation of eating, degeneracy occurs across at least two different scales (71): there are multiple pathways for triggering cessation of eating, (a) suppression of hunger neurons in the arcuate nucleus, (b) delay in gastric emptying, and (c) communication with the dorsal vagal complex through the vagus nerve. In addition, there are multiple independent hormone and receptor combinations that trigger each of these mechanisms (71). Moreover, there are also a variety of time scales, ranging from milliseconds/seconds (neural circuits) over minutes/hours (neuromodulators and gut satiety peptides) to hours/days (adipokines and insulin) (71). In literature, time scores of fullness and hunger have been used as a proxy for the time dependent satiety/satiation level (see e.g., Gibbons et al. (90)) and this can thus be used as quantification proxy (for M4).

Rozin and Rozin (91) argue that humans as omnivores are generalists, species that can eat a wide range of foods and are limited in their food supply primarily by environmental availability and competition with other species. The varied diet (M5) means that humans can eat relatively small amounts of any given food, so that if a particular food is mildly toxic, it will not be harmful because people will not have consumed a large amount. Furthermore, a varied diet produces an increase in the probability of ingestion of all required micronutrients (91). Additionally, the diets of Paleolithic humans must have varied greatly with geographical location and season just as do those of recently studied hunter-gatherers (35, 92). So, from evolution, dietary flexibility is baked in. For instance Henry et al. (93) argue that the lack of dietary flexibility (due to technological and social constraints) may have contributed significantly to the extinction of Neanderthals. A potential proxy here could be the perceived variety of food types by a human panel.

In the Paleolithic environment food availability was more uncertain, energy-dense foods, and calories were harder to acquire (92, 94, 95), and these challenges for survival and successful reproduction likely led to the evolution of adaptive preferences for energy-dense foods—foods that were high in fat, sugar and calories (see M2 above)—and for the capacity to store excess calories as body fat (M6). Lipid storage is an evolved adaptation that allows individuals to continue to survive and reproduce in the face of temporary shortfalls in energy intake from food (95, 96). Maintaining fat stores and energy reserves in general at a level that will maintain life and support reproduction is a strong selection pressure, given the alternating periods of plenty and of shortage. Subsequently, mechanisms to prevent starvation have evolved in complex ways that affect foraging, feeding, sleep patterns, metabolism and energy storage (71). Potentially energy density can be a useful proxy here.

And finally, it is well known that humans are social animals and that our eating is also socially influenced (M7), see for instance (97) for a review discussing social modeling, whereby people use others’ eating as a guide for what and how much to eat. Research suggests that people tend to eat more when eating with other people, compared with when they eat alone, and this is known as the social facilitation of eating (98). Ruddock et al. (98) argues that social facilitation of eating has evolved as an efficient evolutionary adaptation. There seems to be a (culturally ingrained) intuition to correlate healthy with not tasty while unhealthy is associated with tasty (99). A perceived societal acceptance score of food types by a human panel might be an appropriate quantifiable proxy.

Having identified this set of main characteristics of the innate human food intake control systems, we can now map the properties commonly associated with UPFs on to them, and this is done in the next section.

## 4 Proposed integrative conceptual framework to evaluate the interaction of UPFs properties to our evolved food intake regulation mechanism

This section integrates the prior sections by mapping the product features commonly associated with UPFs (Section 2) onto the main characteristics of our evolved food intake regulation mechanisms (Section 3). It is divided in two subsections: in the first we detail the actual framework containing the linkages, whereas in the second part we evaluate the usefulness of the proposed framework in two contexts of reduced appetite for UPFs.

### 4.1 Linking characteristics of ultra-processed foods to evolved food intake regulation mechanisms

As shown in Section 2.2, the properties which are commonly attributed to UPFs to explain their appeal, are not unique to them, even though on average they rate higher on them than non or minimally processed foods. In contrast to the latter, UPFs do combine more of them at the same time, frequently up to 7–9 of the 9 identified properties, whereas non or minimally processed foods might combine at most 2 to 3 (see Supplementary Table S1). In this section we will now evaluate how our food control intake system might be influenced by combinations of the properties. In Table 3, we map the common UPF properties to the main characteristics of the food intake regulation control system we identified in Section 3. We will first describe these hypothesized linkages in more detail and then conclude and propose a path forward.

**Table 3:** Overview of hypothesized linkages between food intake control characteristics and the properties commonly attributed to UPFs.

Food intake regulation system characteristic	Hypothesis for which properties commonly attributed to UPFs have an influence
M1. Drive for efficient foraging	P2 High eating rate, P3 High energy density, P5 High convenience, P6 Low cost, P8 Ubiquitous presence, P9 Long shelf-life
M2. Preference for salt, sweet…	P1 Engineered palatability
M3. Hedonic influence	P1 Engineered palatability P3 High energy density P4 High variety, P7 Highly marketed
M4. Time-lag of satiation/satiety	P2 High eating rate, P3 High energy density, P5 High convenience
M5. Preference for dietary flexibility	P1 Engineered palatability, P4 High variety
M6. Asymmetric energy regulation	P2 High eating rate, P3 High energy density, P5 High convenience, P6 Low cost, P8 ubiquitous presence
M7. Social modulation and social learning	P7 Highly marketed

Considering the drive for efficient foraging (M1), any property that decreases the cost of acquisition and digestion, should have an influence, which explains the hypothesized linkages with P2 High eating rate, P3 High energy density, P5 High convenience, P6 Low cost, P8 Ubiquitous presence and P9 Long shelf-life.

For the innate preference for salt, sweet, … (M2), the engineered palatability of UPFs is geared toward this: UPFs frequently contain high amounts of salt, sugar and fat so that in general UPFs elicit higher approach motivation than unprocessed and minimally processed foods (29).

Coming to our hedonic eating control system (M3), any property that increase the reward value of foods should potentially have an influence, explaining the hypothesized influence of P1 Engineered palatability, P3 High energy density and P4 High variety. Advertisements for UPF are frequently reward driven (P7 Highly marketed) appealing to our hedonic control system (100).

The time-lag for satiation and satiety signals to occur (M4) are influenced by the speed of consumption so that we can surmise that P2 High eating rate, P3 High energy density, and P5 High convenience will all have an influence since they directly increase the consumption rate.

P1 Engineered palatability and P4 High variety are hypothesized to interact with the preference for dietary flexibility (M5), the latter at total consumption level and the former at the individual product level.

Anything that facilitates the intake of calories (i.e., P2 High eating rate, P3 High energy density, P5 High convenience, P6 Low cost, P8 Ubiquitous presence) can be expected to speak to the asymmetric energy regulation (M6) characteristic.

And finally advertisements for UPF frequently urge people to be part of a group (P7 Highly marketed) and thus can be expected to speak to the Social Modulation (M7) characteristic: for instance Ilieva et al. (101) conclude that social norms emerges as a strong determinant for purchase decisions of UPFs based on an analysis of an online questionnaire study.

Upon considering Table 3, a few things can be noted: first all the properties interact with different food intake regulation system characteristics (i.e., 4 for P3, 3 for P1, P2, P5 and 2 for the rest), and the latter are nearly all influenced by multiple properties commonly ascribe to UPFs. Hence, a food that combines multiple or even better all the properties attributed to UPFs is hypothesized to have a greater impact on our food intake control system, then foods that only have 1 or 2 of these properties.

The framework proposed in Table 3 lends itself to experimental evaluation, since an experiment can be set up whereby one compares the impact (using the proxies in Table 2) of foods having different levels of the properties (quantified by the proxies in Table 1).

In the next section we will evaluate the usefulness of the proposed framework to explain two real-life changes in the attractiveness of UPFs.

### 4.2 Using the proposed framework to assess two scenarios of reduced attractiveness of UPFs

To illustrate the potential usefulness of the proposed framework, we evaluate two contexts in which UPFs appeal and intake patterns often shift: bariatric surgery, GLP-1 receptor agonist treatment.

Bariatric surgery results not only in decreased food intake, but also in changes in frequency of food intake (fewer snacks, less food per meal) and in food preference. Post surgery, patients have reduced preference for sweet and fat taste and for high-calorie foods (reduction in M2) (102). Food containing fat and simple carbohydrate may be labeled as “not good” due to negative post ingestive sequelae (affecting M3). This can result in altered food preferences observed after bariatric surgery. The mechanism behind this alteration is postulated to be due to visceral signals and changes in taste function (103). After the operation, people may still “like” sweet and fatty food but they “avoid” eating the same quantity as they did before the operation, possibly in an attempt to avoid negative visceral signals associated with larger quantities (103). In addition research suggests that there is a rearrangement of hormonal and neural elements of gastrointestinal tract, resulting in secondary changes in food intake due to increased satiety and satiation (affecting M4) and appetite suppression (102).

Glucagon-like peptide-1 (GLP-1) receptor agonist (RAs) seem to work by artificially amplifying the satiation/satiety signals (affecting M4). In effect, these agents mimic the effects of endogenous GLP-1, which is produced in the intestines in response to food intake and is quickly enzymatically inactivated (104). Centrally, GLP-1 RAs modulate brain regions controlling appetite and peripherally they delay gastric emptying and regulate gut hormones (104). Apart from decreasing general food cravings and hunger feeling, they also seem to have an influence on food preferences themselves. This is still a developing area of research, but there are some emerging strands: e.g., Awad et al. (105) find, using household scanner data, that the introduction of GLP-1 leads to a statistically significant reduction of approximately $56 per month in total food spending and induces a systematic reallocation of the household food budget away from ultra-processed foods and toward minimally processed foods. Blundell et al. (106) found that once weekly semaglutide administration, led to a lower relative preference for fatty, energy-dense foods (reduction in M2). Recent work by West et al. (107) confirms that these GLP-1 RA medications work in generating significant weight loss, but that once people stop taking them, this is followed by rapid weight regain and reversal of beneficial effects on cardiometabolic markers.

These scenarios suggest that the current framework can be used to conceptually evaluate the impact of UPFs properties on intake in different contexts.

## 5 Conclusion

Human evolution has endowed *Homo sapiens* with a remarkable capacity to adapt and thrive across a wide range of environments (92). In today’s environment of food abundance, however, those same adaptive strategies can become liabilities. This analysis has argued that many ultra-processed foods interact with a set of characteristics of the food intake regulation system, collectively biasing individuals toward higher consumption of UPFs relative to minimally or non-processed foods. UPFs achieve this by combining multiple intake-driving properties (e.g., highly rewarding taste profiles, rapid digestibility, and variety that thwarts sensory satiety) with pervasive availability and convenience. Our mechanistic property-based perspective complements established economic and socio-cultural models: it helps to explain why UPFs are not just prevalent, but uniquely compelling, often overriding internal signals to stop eating. A holistic explanation for the contemporary dominance of ultra-processed foods therefore requires explicit attention to interactions between UPFs properties and characteristics of the food intake regulation system, alongside structural drivers such as pricing, food-system incentives, marketing, and policy.

The current analysis suggests that ultra-processed foods are an extension of a longstanding trajectory of humans modifying their food intake, and not a categorical break. Hence it should come as no surprise that for those properties that are commonly used to explain the attractiveness of UPFs, the latter do not form a separate category separated by clear threshold levels versus minimally or non-processed foods. On average, UPFs score higher on the quantified proxies defined in Table 1, indeed suggesting that they are a further development, rather than a clear departure from historical food processing. The one way that UPFs do clearly differ, is in the fact that they combine (nearly) all the identified properties at the same time, whereas for minimally or non-processed foods these only have two or three at the same time.

A key next step would be to further experimentally evaluate the proposed linkages by manipulating specific UPFs features (e.g., eating rate via matrix structure; sensory variety; cue exposure and branding) while measuring the food intake regulation system characteristics (such as satiation/satiety dynamics) under ecologically valid conditions using the proposed quantified proxies. This would allow the relative contribution of different pathways to be estimated and would clarify which combinations of features constitute the most potent “intake-biasing” configurations.

Because UPFs simultaneously activate multiple reinforcing pathways (reward, eating rate, variety, cue learning, convenience, and affordability), single, isolated policy measures (e.g., taxation alone, advertising restrictions alone, or guideline revisions alone) are unlikely to be sufficient. Rather, multi-level strategies that act on several pathways in parallel, e.g., improving the palatability and convenience of healthier foods even as we reduce the ubiquity and appeal of ultra-processed options, are more likely to shift population diets meaningfully.

At the population level, dietary transitions toward healthier patterns may depend not only on regulatory and economic interventions, but also on making minimally processed or less-processed alternatives more competitive on the very dimensions that matter to evolved food intake regulation mechanisms: sensory satisfaction, convenience, affordability, and ease of consumption. This framing also cautions against attributing obesity or diet-related disease to a single dietary component (e.g., sugar, fat, or carbohydrates). A more productive approach integrates physiology, genetics, behavioral science, psychology, and nutrition while explicitly modeling interactions between biological predispositions and UPFs-rich diets (85).

## Footnotes

1 Note that there is some variation across literature in terms of the estimated amount of ultra-processed foods in the average diet, for various reasons such as lack of consistency in the application of the criteria (see further), variation in the data-set on which the assessment is based and variation of how the relative amount is calculated (e.g., as % of mass consumed, as % of total energy input, …).
