# Supplementary material for: From scarcity to ultra-processed foods: a conceptual analysis of how their properties interact with characteristics of food intake regulation mechanism
Source: Front Nutr. 2026 Apr 10;13:1803885. doi: 10.3389/fnut.2026.1803885 (PMC13105965; doi:10.3389/fnut.2026.1803885)
Supplement: Supplementary file 1 [file Table_1.docx]

Supplementary Material

**Supplementary Table S1: mapping of which main characteristics of UPFs identified in section 2.2 in Figure 1 are relevant for which categories of UPFs and for minimally and non-processed foods (here we take the categories from (20)).**

|  |  | P1. Engineered palatability | P2. High eating rate/soft texture | P3. High energy density | P4. High sensory variety, contrast and novelty | P5. Convenience / low friction | P6. Economic accessibility and price engineering | P7. Branded, marketed, | P8. Ubiquity and strategic availability/placement | P9. Shelf-life/safety/standardization |
| --- | --- | --- | --- | --- | --- | --- | --- | --- | --- | --- |
| Ultra-processed food categories | industrial breads | ✅ | ✅ | ✅ | ✅ | ✅ | ✅ | ✅ | ❌ | ✅ |
|  | frozen/shelf-stable dishes | ✅ | ✅ | ✅ | ✅ | ✅ | ✅ | ✅ | ❌ | ✅ |
|  | confectionery | ✅ | ✅ | ✅ | ✅ | ✅ | ✅ | ✅ | ✅ | ✅ |
|  | fruit and milk drinks | ✅ | ✅ | ❌ | ✅ | ✅ | ✅ | ✅ | ❌ | ✅ |
|  | cakes, cookies and pies | ✅ | ✅ | ✅ | ✅ | ✅ | ✅ | ✅ | ✅ | ✅ |
|  | soft drinks | ✅ | ✅ | ❌ | ✅ | ✅ | ✅ | ✅ | ✅ | ✅ |
|  | salty snacks | ✅ | ✅ | ✅ | ✅ | ✅ | ✅ | ✅ | ✅ | ✅ |
|  | breakfast cereals | ✅ | ✅ | ✅ | ✅ | ✅ | ✅ | ✅ | ❌ | ✅ |
|  | sauces, dressings and gravies | ✅ | ✅ | ✅ | ✅ | ✅ | ✅ | ✅ | ❌ | ✅ |
|  | sausages, hamburgers, reconstituted meat products | ✅ | ✅ | ✅ | ✅ | ✅ | ✅ | ✅ | ✅ | ✅ |
| Minimally or non-processed categories | cereal | ❌ | ❌ | ❌ | ❌ | ❌ | ❌ | ❌ | ❌ | ❌ |
|  | meat | ❌ | ❌ | ❌ | ✅ | ❌ | ❌ | ❌ | ❌ | ❌ |
|  | milk | ❌ | ✅ | ❌ | ❌ | ✅ | ❌ | ❌ | ❌ | ✅ |
|  | poultry | ❌ | ❌ | ❌ | ✅ | ❌ | ❌ | ❌ | ❌ | ❌ |
|  | fruits | ❌ | ❌ | ❌ | ✅ | ✅ | ❌ | ❌ | ❌ | ❌ |
|  | roots and tubers | ❌ | ❌ | ❌ | ❌ | ❌ | ❌ | ❌ | ❌ | ❌ |
|  | eggs | ❌ | ❌ | ❌ | ❌ | ❌ | ❌ | ❌ | ❌ | ❌ |
|  | legumes | ❌ | ❌ | ❌ | ❌ | ❌ | ❌ | ❌ | ❌ | ❌ |
|  | fish | ❌ | ❌ | ❌ | ❌ | ❌ | ❌ | ❌ | ❌ | ❌ |
|  | vegetables | ❌ | ❌ | ❌ | ❌ | ❌ | ❌ | ❌ | ❌ | ❌ |
